# Increased Appetite Plays a Key Role in Olanzapine-Induced Weight Gain in First-Episode Schizophrenia Patients

**DOI:** 10.3389/fphar.2020.00739

**Published:** 2020-05-22

**Authors:** Jing Huang, Gang-Rui Hei, Ye Yang, Chen-Chen Liu, Jing-Mei Xiao, Yu-Jun Long, Xing-Jie Peng, Yi Yang, Jing-Ping Zhao, Ren-Rong Wu

**Affiliations:** ^1^Mental Health Institute of the Second Xiangya Hospital, Central South University, Changsha, China; ^2^China National Clinical Research Center on Mental Disorders, Changsha, China; ^3^China National Technology Institute on Mental Disorders, Hunan Key Laboratory of Psychiatry and Mental Health, Changsha, China; ^4^Mental Health Institute, Wenzhou Kangning Hospital, Wenzhou Medical University, Wenzhou, China; ^5^Shanghai Institutes for Biological Sciences, Chinese Academy of Sciences, Shanghai, China

**Keywords:** antipsychotic drugs, appetite, olanzapine, schizophrenia, weight gain

## Abstract

Weight gain and metabolic disturbances, potentially influenced by increased appetite, are common effects of olanzapine treatment in patients with schizophrenia. In this study, we explored the association between olanzapine-induced weight gain and metabolic effects with increased appetite. Drug-naïve, first-episode schizophrenia patients were treated with olanzapine for 12 weeks. Assessments included time to increased appetite, body weight, body mass index, biochemical indicators of blood glucose and lipids, proportion of patients who gained more than 7% or 10% of their baseline weight upon treatment conclusion, patients who developed dyslipidemia, and Positive and Negative Syndrome Scale scores. In total, 33 patients with schizophrenia receiving olanzapine were enrolled and 31 completed the study. During the 12-week olanzapine treatment, 77.4% (24/31) patients had increased appetite with 58.1% (18/31) patients having increased appetite within the first 4 weeks. The mean time for increased appetite was 20.3 days. More patients in the increased appetite group increased their initial body weight by more than 7% after 12 weeks when compared to patients with unchanged appetite (22/24 [91.7%] vs. 3/7 [42.9%], p = 0.004). Earlier increased appetite led to more weight gain during the following month. Overall, 50% of patients in the increased appetite group had dyslipidemia after 12 weeks. Our results demonstrated that olanzapine induced significantly appetite increase in first-episode patients with schizophrenia and appetite increase played a key role in olanzapine-induced weight gain and dyslipidemia.

**Clinical Trial Registration:**

NCT03451734. Registered March 2, 2018 (retrospectively registered).

## Introduction

Olanzapine is one of the most widely used second-generation antipsychotics (SGAs) for schizophrenia, bipolar disorder, and psychotic symptoms. Besides improving the main symptoms of psychosis, olanzapine shows great acceptability, decreases all-cause discontinuation, and prevents future relapse (Leucht et al., 2009). Many randomized clinical trials (RCTs) and meta analyses have suggested that olanzapine is one of the most efficacious antipsychotic drugs in patients with schizophrenia (Leucht et al., 2013; Huhn et al., 2019).

The most common adverse effects of olanzapine is weight gain (Allison et al., 1999). Olanzapine elicits the most weight gain of the SGAs. The Comparison of Atypicals for First Episode (CAFE) trial demonstrated that 80% of patients treated with olanzapine gained more than 7% of their initial body weight at week 52 (McEvoy et al., 2007). The discontinuation rate of olanzapine due to weight gain or accompanying metabolic effects was 2–8 times higher than other antipsychotics in the Clinical Antipsychotic Trials in Intervention Effectiveness (CATIE) trial (Lieberman et al., 2005). Besides being associated with decreased adherence with drug treatment, weight gain is also associated with substantial medical morbidity and mortality. Considering the high obesity rate in patients with schizophrenia (42%), the potential risk of olanzapine-induced weight gain should be evaluated carefully (Newcomer, 2006). Some randomized controlled trial (RCT) studies have found that patients with mental diseases die up to 30 years earlier than the general population (Das-Munshi et al., 2017). The leading cause of death in this population is heart disease. A major risk factor for heart disease and premature death in these patients is weight gain (Fekadu et al., 2015).

The mechanisms underlying antipsychotic-induced weight gain and adverse metabolic effects are not well understood (Correll et al., 2011). Olanzapine is associated with elevated appetite and food intake and decreased activity or impairment of metabolic regulation (Roerig et al., 2005; Henderson et al., 2015). Fountaine et al. reported that in healthy men, olanzapine increased body weight through increased food intake, without evidence of decreased activity or expenditure levels (Fountaine et al., 2010). This observation is in agreement with observations of male adolescent inpatients with schizophrenia (Gothelf et al., 2002). Patients had significantly increased body mass index due to increased caloric intake after 4-week olanzapine treatment (Gothelf et al., 2002). However, current evidence regarding the association of appetite with weight gain is inconclusive (Poyurovsky et al., 2007; Case et al., 2010). Case et al. reported that early appetite changes were not consistently correlated to overall weight change in four different trials (Case et al., 2010). Further, no study has reported the exact time of appetite increase, compared weight gain velocity relative to the timing of increased appetite, or assessed differences in weight gain and metabolic effects between patients with increased or unchanged appetite. Therefore, we evaluated the association between appetite increase and olanzapine-induced weight changes and accompanying metabolic effects in drug-naïve first-episode patients with schizophrenia.

## Materials and Methods

### Participants

This study was conducted in the Mental Health Institute of the Second Xiangya Hospital, Central South University, China between December 2016 and April 2019. Participants were assessed for schizophrenia in accordance with criteria defined by the Diagnostic and Statistical Manual of Mental Disorders-Fifth Edition (DSM-5) (A.P. Association, 2013). As antipsychotic-naïve/first episode patients appear to gain more weight after olanzapine treatment (Correll et al., 2011), we included first-episode schizophrenia patients aged 18–50 years were included in this study.

The exclusion criteria included (i) clinically abnormal findings of physical examinations, laboratory tests, or electrocardiogram (ECG) results; (ii) disorders such as intellectual disability, substance or alcohol use disorder, a diagnosis of other specific systemic diseases according to DSM-5 criteria; (iii) cardiovascular and metabolic diseases such as diabetes mellitus, dyslipidemia, and hypertension; (iv) a history of eating disorders; (v) strict diet within the month before screening or during the study; and (vi) pregnancy or lactating.

### Intervention

Previous studies have suggested that the rate of olanzapine-induced weight gain was most rapid during the first 12 weeks of treatment (Correll et al., 2009). Therefore, participants were treated with olanzapine (15–20 mg/day at 8:00 p.m.) for 12 weeks. The initial dose of olanzapine was 5 mg/day and then adjusted to 15–20 mg/day in the first week.

### Assessment

Baseline assessments included demographics, a thorough medical history, anthropometric measurements (weight and height), appetite, physical examination, and lab analysis. Appetite was assessed daily, 30 min before lunch, with four standardized questions: Hungry, Felt full, Thinking about food, and Overeating. Responses were graded on a scale from 0 to 10, where 0 = “not at all” and 10 = “extremely”. Appetite increase was defined as a >10% increases in baseline appetite scores. Appetite decrease was defined as a >10% decreases in baseline appetite scores. The Positive and Negative Syndrome Scale (PANSS) was used to evaluate the severity of schizophrenia symptoms. Adverse effects were evaluated by Treatment Emergent Symptom Scale (TESS). At each follow-up visit, all baseline evaluations (including physical examination, anthropometric measurements, appetite, and TESS) were repeated except PANSS. PANSS was re-evaluated at week 12.

Prior to treatment and in the fasting state, weight and height were measured after participants removed shoes with light indoor clothing. Appetite was assessed before lunch and was based on the judgement of the rating physician on examination day. Lab work including plasma glucose, liver, and renal function were evaluated using enzymatic procedures with the Boehringer Mannheim/Hitachi 714 automated chemistry analyzer. Insulin was measured with a solid-phase enzyme-linked immunosorbent assays (ELISA).

The primary outcomes were the percentage of patients who had increased appetite and period of time between olanzapine treatment and increased appetite. Secondary outcomes included changes in weight, body mass index (BMI), fasting glucose, fasting insulin and insulin resistance index, lipid profiles which included triglycerides, cholesterol, high-density lipoprotein (HDL-C), and low-density lipoprotein (LDL-C), and PANSS score. BMI was calculated according to the criteria of the Working Group on Obesity in China: healthy weight (18.5 ≤ BMI <25 kg/m^2^), overweight (25 ≤ BMI ≤ 28 kg/m^2^), and obese (BMI > 28 kg/m^2^). Dyslipidemia was defined as cholesterol ≥ 5.18 mmolċl^−1^, triglycerides ≥ 1.70 mmolċl^−1^, HDL-C < 1.04 mmolċl^−1^, or LDL-C ≥ 3.37 mmolċl^−1^ based on the Chinese guidelines for dyslipidemia (Hu, 2017). An analysis of the proportion of patients who gained more than 7% of their baseline body weight after 12 weeks, which is the cutoff for clinically significant weight gain, was also included (Kanders et al., 1991).

### Statistical Analysis

Statistical Package for Social Sciences, version 25.0 (SPSS v25.0) was employed for statistical analysis. Continuous variables and categorical variables are described using summary statistics (means and standard deviations) or frequencies and percentages, respectively. Student's t-test and chi square analyses were used to analyze between-group differences in changes of body weight, BMI, fasting glucose and insulin, insulin resistance index, triglyceride, cholesterol, HDL-C, and LDL-C from baseline to each time point. We investigated the association between weight, BMI, insulin resistance index, LDL-C, and appetite using linear regression analysis. A P-value (p) < 0.05 was considered statistically significant.

## Results

In total, 33 schizophrenia inpatients (mean age, 23.5 years; range 18–36 years) were enrolled in the study. There was a higher proportion of female patients (63.6%, 21/33). All patients were in the normal BMI range (mean BMI, 21.3 ± 1.7 kg/m^2^). The mean duration of schizophrenia was 11.2 ± 3.7 (range 5–18) months. Two female patients dropped out of the study after 4 weeks because of increased appetite; the remaining 31 (93.9%) participants completed the study (**Table 1**).

**Table 1:** Summary of weight and metabolic measures by study.

Variable and Week	Mean	SD	adjusted P Value
**Weight** (kg)			
Week 0	56.69	8.75	
Week 4	59.05	8.52	<0.001
Week 8	63.54	8.09	<0.001
Week 12	65.07	8.11	<0.001
**Body mass index** (kg/m^2^)			
Week 0	21.33	1.72	
Week 4	22.24	1.64	<0.001
Week 8	23.92	1.67	<0.001
Week 12	24.51	1.84	<0.001
**Fasting glucose** (mmol l^–1^)			
Week 0	4.39	0.36	
Week 4	4.48	0.40	0.3460
Week 8	4.57	0.34	0.0338
Week 12	4.60	0.33	0.0020
**Fasting insulin** (mIU l^–1^)			
Week 0	7.81	1.74	
Week 4	8.38	2.08	0.2841
Week 8	10.57	3.28	<0.001
Week 12	13.94	5.46	<0.001
**Insulin resistance index**			
Week 0	1.53	0.39	
Week 4	1.67	0.44	0.2219
Week 8	2.15	0.71	<0.001
Week 12	2.85	1.13	<0.001
**Triglyceride** (mmol l^–1^)			
Week 0	0.73	0.27	
Week 4	0.88	0.33	0.0066
Week 8	1.09	0.44	<0.001
Week 12	1.27	0.55	<0.001
**Cholesterol** (mmol l^–1^)			
Week 0	3.73	0.48	
Week 4	3.61	0.48	0.1811
Week 8	3.89	0.55	0.1511
Week 12	4.17	0.77	0.0022
**HDL-C** (mmol l^–1^)			
Week 0	1.22	0.13	
Week 4	1.22	0.15	0.9939
Week 8	1.17	0.16	0.1412
Week 12	1.09	0.25	0.0134
**LDL-C** (mmol l^–1^)			
Week 0	2.10	0.35	
Week 4	2.12	0.39	0.9972
Week 8	2.43	0.51	0.0039
Week 12	2.59	0.61	<0.001

HDL-C, high-density lipoprotein cholesterol; LDL-C, low-density lipoprotein cholesterol.

### Appetite Increase After Olanzapine Treatment

**Figure 1:**
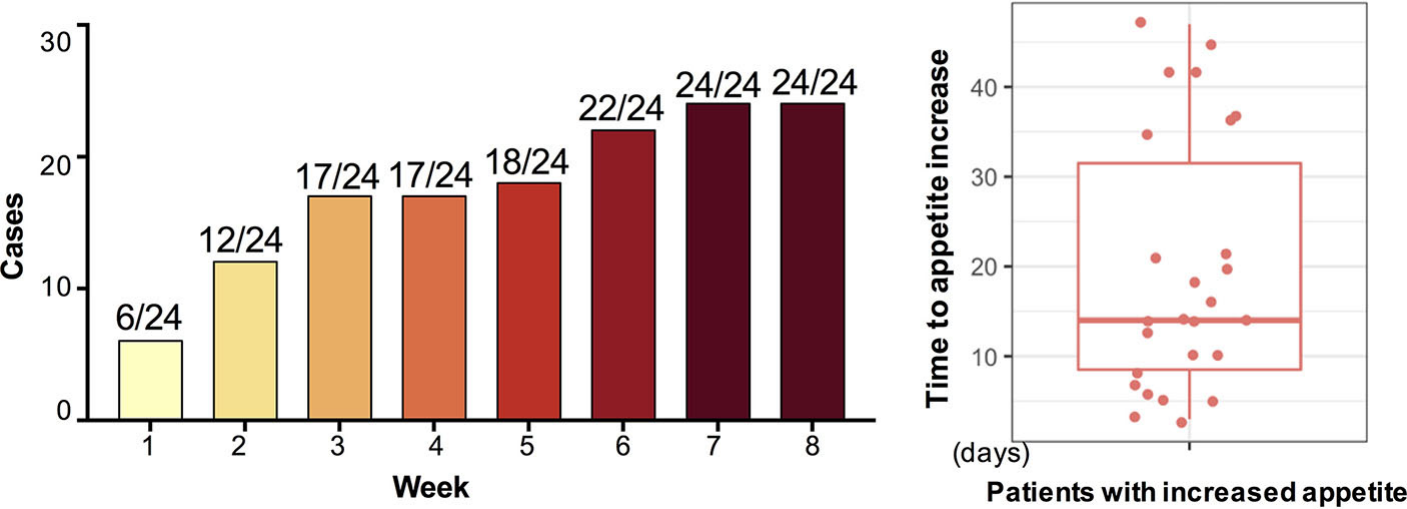
Number of patients with increased appetite during 8-week olanzapine treatment periods.

After 12-week olanzapine treatment, 77.4% (24/31) patients had increased appetite and 22.6% (7/31) patients had unchanged appetite. As shown in **Figure 1**, for the 24 patients who had increased appetite, the appetite increase began within the first 8 weeks of olanzapine treatment and lasted until the end of treatment. The mean time from olanzapine treatment initiation to appetite increase was 20.3 days (SD = 14.4), and 25.0% (6/24) and 70.8% (17/24) increased their appetite within 1 week and 4 weeks, respectively. Two patients increased their appetite on the 3^rd^ day after olanzapine treatment. No significant difference was found in olanzapine-induced appetite between different genders using two-way analysis ANOVA.

### Changes of Body Weight and BMI After Olanzapine Treatment

Significant increases in weight and BMI were observed after olanzapine treatment (**Table 1**). The mean weight gain was 7.9 kg during the 12-week period of olanzapine treatment, with patients gaining 2.4, 4.0, and 1.5 kg at the first, second, and third 4-week period, respectively. Female patients were more likely to gain weight (P < 0.001), with 9.19-kg mean weight gain during the study period compared with 5.90-kg weight gain in male patients. Of the total patients, 80.6% (25/31) and 61.3% (19/31) increased their initial body weight by more than 7% and 10%, respectively, during the 12-week olanzapine treatment (**Supplementary Table 1**). After 12 weeks, 51.6% (16/31) patients became overweight (BMI>25 kg/m^2^).

### Changes of Metabolic Disturbances After Olanzapine Treatment

Significant increases in fasting glucose, fasting insulin, insulin resistance index, triglyceride, cholesterol, and LDL-C and decreases in HDL-C were observed at 12 weeks (**Table 1**). No patient included in the study had dyslipidemia at baseline. Based on Chinese guidelines for dyslipidemia, 38.7% (12/31) of patients had dyslipidemia after 12 weeks of olanzapine treatment.

### Appetite Increase and Olanzapine Induced Weight Gain

In order to detect the effect of increased appetite on olanzapine-induced weight gain, we divided the patients into two groups (increased appetite group [n = 24] and unchanged appetite group [n = 7]) based on whether the appetite increased or not after 12 weeks of olanzapine treatment.

**Figure 2:**
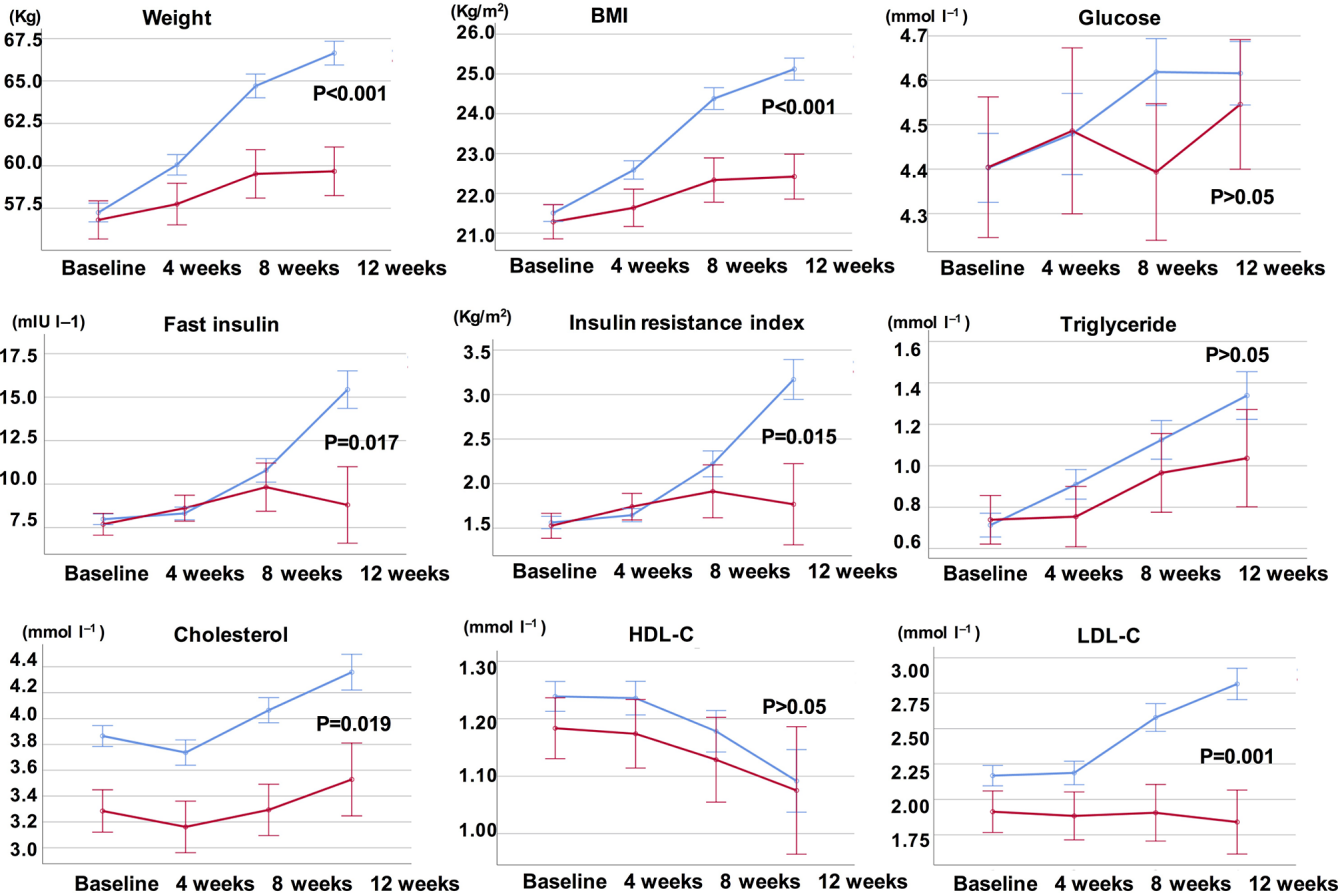
Comparison of body weight, BMI, glucose, and lipid levels between increased and unchanged appetite groups. Blue line indicates patients with increased appetite; red line indicates patients with unchanged appetite.

Compared with the unchanged appetite group, there were significant increases in weight and BMI levels in the appetite increase group after 12-week olanzapine treatment (**Table 2**). **Figure 2** showed the comparison of weight gain and metabolic-related outcomes between increased and unchanged appetite groups. Detailed analyses were presented in **Supplementary Table 2**. The weight gain between two groups was significant (P = 0.01) using two-way analysis ANOVA. The mean weight increased by 9.1 kg (SD = 4.1) in the appetite-increased group and 3.9 kg (SD = 2.0) in the unchanged appetite group with significant difference between the two groups during 12-week olanzapine treatment period. A greater percentage of patients (91.7%) in the increased appetite group gained more than 7% of their initial body weight after 12 weeks when compared to patients in the appetite unchanged group (42.9%; χ^2^ = 8.27, df = 1, p = 0.004). Similarly, more patients in the increased appetite group (19/24, 79.2%) gained more than 10% of initial body weight after 12 weeks when compared to patients in the unchanged appetite group (0.0%; χ2 = 14.32, df = 1, p < 0.001) (**Supplementary Tables 3**–**5**). After 12-week olanzapine treatment, 58.3% patients became overweight in the appetite increased group compared to 28.6% of patients in the unchanged appetite group. Appetite increase strongly mediated olanzapine-induced weight gain with mediating effect 66.2%.

**Table 2:** Descriptive statistics of outcome measures between increased and unchanged appetite groups at baseline, and weeks 4, 8, and 12.

	Baseline			Week 4			Week 8			Week 12			*P*-value	Sig
	N	Mean	s.d.	N	Mean	s.d.	N	Mean	s.d.	N	Mean	s.d.		
**Weight** (kg)														
Appetite increased	26	55.06	8.19	20	57.90	8.06	24	62.79	7.55	24	64.62	7.77	<0.0001	***
Appetite unchanged	7	62.76	8.62	13	60.82	9.23	7	66.09	9.92	7	66.61	9.66	<0.0001	***
**Body mass index** (kg/m2)														
Appetite increased	26	21.09	1.74	20	22.23	1.68	24	24.07	1.67	24	24.78	1.84	<0.0001	***
Appetite unchanged	7	22.24	1.42	13	22.26	1.66	7	23.39	1.70	7	23.59	1.62	<0.0001	***
**Fasting glucose** (mmol l–1)														
Appetite increased	26	4.35	0.33	18	4.52	0.43	24	4.59	0.36	24	4.58	0.34	0.0006	***
Appetite unchanged	7	4.53	0.45	13	4.42	0.36	7	4.49	0.27	7	4.69	0.30	0.6509	ns
**Fasting insulin** (mIU l^–1^)														
Appetite increased	26	7.58	1.62	18	8.14	1.73	24	10.53	3.18	24	15.13	5.53	<0.0001	***
Appetite unchanged	7	8.64	2.03	13	8.72	2.52	7	10.71	3.88	7	9.86	2.59	0.2694	ns
**Insulin resistance index**														
Appetite increased	26	1.47	0.34	18	1.63	0.34	24	2.16	0.70	24	3.08	1.15	<0.0001	***
Appetite unchanged	7	1.76	0.51	13	1.72	0.56	7	2.13	0.80	7	2.06	0.56	0.4042	ns
**Triglyceride** (mmol l^–1^)														
Appetite increased	26	0.72	0.27	18	0.94	0.36	24	1.14	0.48	24	1.34	0.59	<0.0001	***
Appetite unchanged	7	0.76	0.30	13	0.78	0.28	7	0.92	0.28	7	1.02	0.34	0.0187	*
**Cholesterol** (mmol l^–1^)														
Appetite increased	26	3.84	0.48	18	3.77	0.48	24	4.04	0.50	24	4.38	0.70	0.00001	***
Appetite unchanged	7	3.32	0.21	13	3.37	0.40	7	3.39	0.45	7	3.47	0.57	0.5206	ns
**HDL-C** (mmol l^–1^)														
Appetite increased	26	1.24	0.13	18	1.23	0.15	24	1.17	0.19	24	1.09	0.29	0.0087	**
Appetite unchanged	7	1.18	0.13	13	1.21	0.15	7	1.14	0.06	7	1.08	0.03	0.0241	*
**LDL-C** (mmol l^–1^)														
Appetite increased	26	2.15	0.35	18	2.07	0.38	24	2.56	0.46	24	2.77	0.55	<0.0001	***
Appetite unchanged	7	1.92	0.29	13	2.19	0.42	7	1.98	0.46	7	2.00	0.38	0.8391	ns

HDL-C, high-density lipoprotein cholesterol; LDL-C, low-density lipoprotein cholesterol. * presents P < 0.05, **presents P < 0.01, ** presents P < 0.001.

### Appetite Increase and Olanzapine Induced Metabolic Disturbances

There were significant differences in insulin (p = 0.017), insulin resistance index (p = 0.015), and LDL-C (p = 0.001) between the appetite increase and unchanged groups after 12 weeks (**Figure 2** and **Table 2**). Four patients in the increased appetite group had cholesterol ≥ 5.18 mmolċl^−1^, nine had triglycerides ≥ 1.70 mmolċl^−1^, six had HDL-C < 1.04ċmmol l^−1^, and three had LDL-C ≥ 3.37 mmolċl^−1^ at week 12. In total, 13/24 patients in the increased appetite group had dyslipidemia after 12 weeks, while none of the patients in the unchanged appetite group developed dyslipidemia during the study period (**Table 3**).

**Table 3:** Dyslipidemia defined by each single outcome measurement in appetite increased and unchanged groups.

	Appetite unchanged		Appetite increased		Chi-Sq	P-value
	N	%	N	%		
Total sample	7		24			
**Total cholesterol** (≥5.18 mmol l–1)	0	0	4	16.7	1.34	0.247121
**Triglyceride** (≥1.70 mmol l–1)	0	0	9	37.5	3.7	0.05445
**HDL-C** (<1.04 mmol l–1)	0	0	6	25	2.17	0.140726
**LDL-C** (≥3.37 mmol l–1)	0	0	3	12.5	0.97	0.324992

### The Peak Weight Gain Reached 1 Month After the Month of Appetite Increase

**Figure 3:**
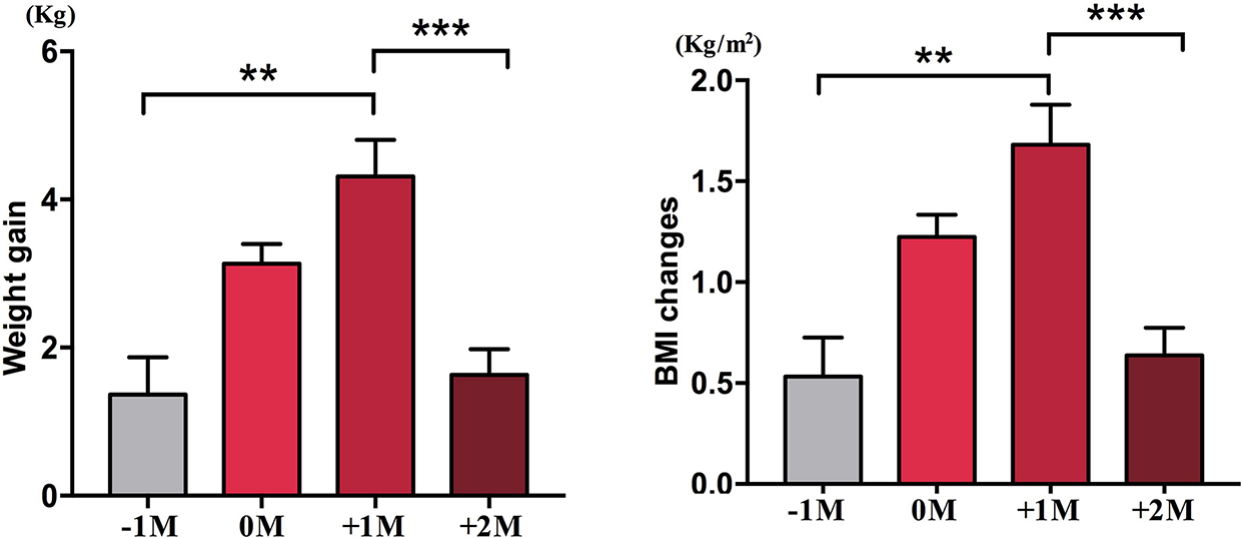
Mean weight gain velocity in increased appetite group. The mean weight gain for each patient per month during the study period was analyzed at four time points: before the month of time to increased appetite (−1M), the month of time to increased appetite (0M), 1 month after the month of time to increased appetite increase (+1M), and 2 months after the month of time to increased appetite (+2M). ** indicates P < 0.01, *** indicates P < 0.001.

In the increased appetite group, we analyzed the mean weight gain for each patient at four time points: before the month of time to appetite increase (−1M), the month of time to appetite increase (0M), one month post-appetite increase (+1M), and two months post-appetite increase (+2M). Compared with −1M, there was an increase in weight gain at 0M, but this change was not significant (−1M vs. 0M, 1.4 ± 1.2 kg vs. 3.1 ± 1.4 kg). Weight gain entered peak growth at +1M, with a 4.3 ± 2.4 kg increase. Weight Growth decelerated at +2M (p < 0.001) by 1.6 ± 1.5 kg. The weight gain at +2M slowed down and stabilized, and no significant differences were observed when compared with −1M (**Figure 3** and **Supplementary Table 6**).

### Earlier Increased Appetite Predicts More Weight Gain in 1 Month After the Month of Appetite Increase

**Figure 4:**
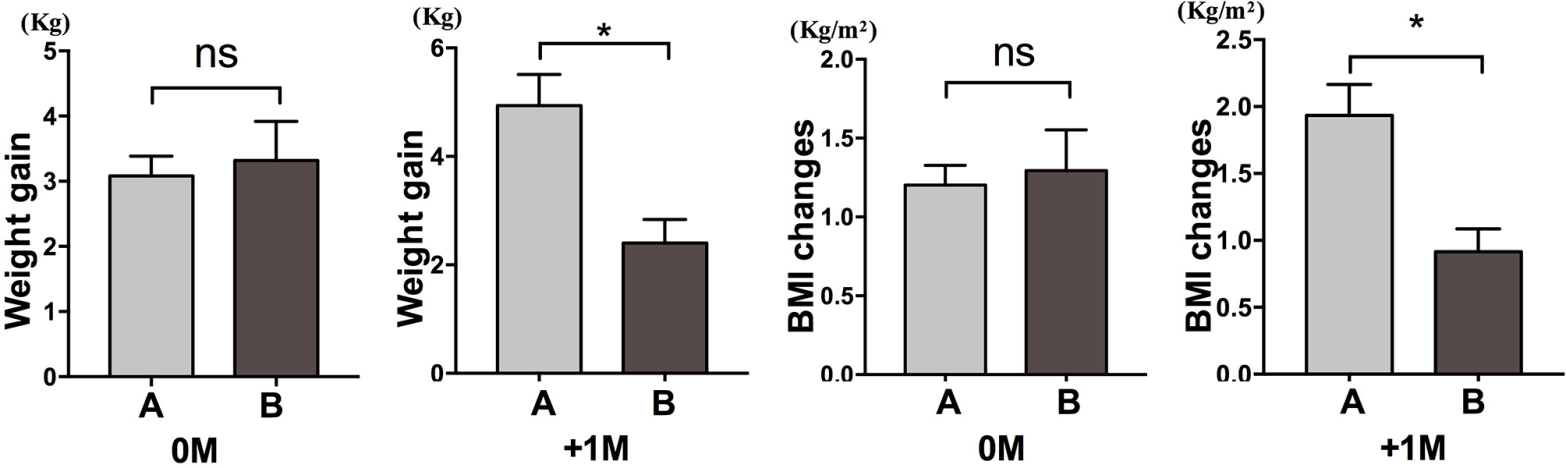
Comparison of weight gain velocity of participants with increased appetite in different months. We compared the weight gain velocity of participants with increased appetite within 4 weeks (A) and participants with an increased appetite between 4 and 8 weeks (B) in the month of time to increased appetite (0M) and 1 month after the month of time to increased appetite (+1M). ns, indicates not significant, * indicates P < 0.05.

We further compared the weight gain velocity of participants with increased appetite within 4 weeks (A) and participants with an increased appetite between 4 and 8 weeks (B). At 0M, the weight gain velocities between participants with increased appetite within 4 weeks and participants with an increased appetite between 4 and 8 weeks were not significantly different. However, participants with earlier increased appetite showed significantly increased weight gain velocity at +1M. These results demonstrated that earlier increased appetite might lead to more weight gain during the follow-up period (**Figure 4**). Similar changes in BMI were observed during the four time points (**Figures 3** and **4**).

### Appetite and Velocity of Metabolic Disturbance Changes

Detailed descriptive data for changes in weight, BMI, fasting glucose, insulin, insulin resistance index, triglyceride, cholesterol, HDL-C, and LDL-C are summarized in **Table 2** To investigate the association between increased appetite and glucose and lipid metabolism, we analyzed changes in these indicators at four time points in the increased appetite group. No significant velocity changes were observed for glucose, triglyceride, HDL-C, and LDL-C at any time point. Significant increases in velocities of insulin and insulin resistance index were only observed at +2M (**Supplementary Figure 1** and **Supplementary Table 6**). Further comparisons suggested that participants with increased appetite within 4 weeks did not show significantly increased velocity of insulin and insulin resistance index at 0M and +1M compared to participants with increased appetite between 4 and 8 weeks (**Supplementary Figure 2**).

### The Prediction of Appetite on Olanzapine-Induced Weight Gain and Metabolic Disturbances

We conducted linear regression analysis to evaluate the effect of appetite increase on weight gain, blood glucose, and lipid levels. After controlling for statistically significant variables such as sex, age, height, and duration, we included weight gain, BMI, fasting glucose, insulin, insulin resistance index, triglycerides, cholesterol, HDL-C, and LDL-C as dependent variables and appetite as the independent variable in the regression analysis. Appetite increase was associated with changes in weight (*β* = 0.67, p =0.0003), BMI (*β* = 0.63, p = 0.0004), insulin (*β* = 0.49, p = 0.019), insulin resistance index (*β* = 0.51, p = 0.0149), and LDL-C (*β* = 0.61, p = 0.0035) (**Supplementary Table 7**).

Significant baseline-to-end point improvements of clinical symptoms (evaluated by PANSS, p < 0.001) were observed after olanzapine treatment. No significant difference in PANSS was observed between the increased appetite and unchanged appetite groups (p > 0.05).

### Adverse Effects

Among all the 31 patients who completed the study, nine (29.0%) patients reported hypoactivity, eight (25.8%) patients reported somnolence, four (12.9%) patients had abnormal liver test, two (6.5%) patients reported akathisia, four (12.9%) patients had constipation, one (3.2%) patient had diarrhea, and two (6.5%) patients felt dizzy, and 80.6% (25/31) patients increased their body weight by more than 7% during the study period.

## Discussion

In this prospective study, the main findings showed that 77.4% patients increased their appetite after olanzapine treatment, and these patients who increased their appetite gained more weight than those patients with unchanged appetite (9.1 kg vs. 3.9 kg). Similarly, more patients in the increased appetite group increased their initial body weight by more than 7 or 10%, suggesting that increased appetite is associated with substantial weight gain in drug-naive first-episode schizophrenia patients treated with olanzapine. Moreover, linear regression analysis also supported that appetite increase was strongly related with olanzapine-induced weight gain. These findings were in accordance with previous reports (Gothelf et al., 2002; Cope et al., 2005). Murashita et al. (2005) observed increased appetite-stimulating ghrelin levels and body fat percentage in schizophrenia patients treated with olanzapine. Results from a randomized double-blind study suggested that olanzapine induced food craving and binge eating to a greater extent than clozapine (Murashita et al., 2005; Kluge et al., 2007). Similar results were also reported in mice treated with olanzapine (Cope et al., 2005). The action of olanzapine on multiple receptor sites, especially the D_2_ and 5H_3_ receptors, which modulate appetite, has also been applied in the treatment of anorexia nervosa and chemotherapy-induced nausea and vomiting (Kluge et al., 2007; Tan et al., 2009; Kafantaris et al., 2011).

We also found that 70.8% patients increased their appetite within the first 4 weeks of initial olanzapine treatment with a mean of 20.3 days to increase. Some patients even increased their appetite as early as the third day after olanzapine treatment. Interesting, patients whose appetites were increased earlier in time were likely to gain more weight than patients whose appetites were increased later in time, and weight gain peaked at 1 month after increased appetite occurred. Although no significant difference was found in olanzapine-induced appetite between male and female, female patients were more likely to gain weight (P < 0.001). Therefore, appetite should be considered as an indicator for predicting olanzapine-induced weight gain, especially in female patients. In clinical settings, doctors should pay more attention to patients whose appetites increase early after olanzapine treatment and should start a plan to prevent weight.

Olanzapine-treated patients exhibited an increase of more than 7% of initial body weight, which is consistent with previous findings (Gothelf et al., 2002; Wu et al., 2008). In addition to significant weight gain, patients with olanzapine treatment had significantly perturbed glucose and lipid metabolism after 12 weeks, which is consistent with previously published observations in patients with schizophrenia (Correll et al., 2011). Compared to patients in the unchanged appetite group, the increased appetite group had significant lipid abnormalities. Fasting glucose was not significantly different between the two groups. Participants with early increased appetite did not show increased velocity of insulin and insulin resistance index in the following months after increased appetite. It is possible that glucose levels are independent of weight gain, as appetite did not affect glucose levels.

Adverse physical health outcomes associated with antipsychotics, such as weight gain, metabolic disturbances, and related morbidity, have long been recognized (Wu et al., 2016). Previous studies have suggested that appetite may predict olanzapine-induced weight gain and adverse metabolic effects (Gothelf et al., 2002; Poyurovsky et al., 2007; Fountaine et al., 2010). To our knowledge, this is the first study to assess the association between appetite, weight gain, and metabolic disturbances after olanzapine treatment in drug-naive first-episode schizophrenia patients. Some findings give us more evidences about how to choose medication reasonably in clinic work and give more benefits for preventing olanzapine-induced weight gain and metabolic disturbances.

There are several limitations to our study. First, this study did not measure food intake such as meals and snacks, although all patients included were provided with the same standardized food menu. High fat and fructose intake may decrease appetite control by affecting central appetite regulation. Indeed, high-fructose diets have adverse effects on central appetite signaling and cognitive function (Lowette et al., 2015; Dalvi et al., 2017). Secondly, we didn't examine leptin and ghrelin levels during the study period. Secreted from adipose tissue and stomach, respectively, leptin and ghrelin play crucial roles in the regulation of food intake and energy metabolism (Cui et al., 2017). Several studies found increased leptin level and decreased ghrelin level in the first few weeks after initiation of olanzapine therapy (Basoglu et al., 2010; Stip et al., 2012; Lu et al., 2015). The increase of leptin level remains stable for several weeks, but ghrelin level increases in the longer period (Sentissi et al., 2008). Further researches should monitor the leptin and ghrelin responses to appetite increase, as well as their relationships with metabolic parameters and clinical effects. In addition, we did not monitor activity levels during the study period. Although some studies reported that olanzapine increased body weight solely by increasing appetite and food intake, with no significant differences in resting energy expenditure (Gothelf et al., 2002; Fountaine et al., 2010). A number studies reported reduced physical activities with olanzapine medication (Perez-Cruzado et al., 2018). In our study, 29.0% patients reported hypoactivity and 25.8% patients reported somnolence during the 12-week olanzapine treatment period. Hillebrand et al. (2005) found that olanzapine treatment reduced physical physical activity in rats exposed to activity-based anorexia. Olanzapine treatment also reduced activity levels of patients with anorexia nervosa, without significant body weight and plasma leptin levels differences compared with untreated patients. Our previous study demonstrated lifestyle interventions, which included psychoeducational, dietary, and exercise programs, can reduce antipsychotic-induced weight gain (Wu et al., 2008). Moreover, it would be better to compare more patients with healthy controls of similar age, which can help to interpret olanzapine-induced appetite increase and metabolic changes are specifically related to its pharmacological properties. Further studies should focus on the mechanisms of increased appetite after olanzapine treatment (Koopmann et al., 2012; Sweeney et al., 2017; Mancuso et al., 2019). The reward system in striatal regions may be associated with antipsychotic-associated weight gain (Nielsen et al., 2016). Altered activity in the subgenual anterior cingulate cortex may also partly underlie increased appetite after olanzapine treatment (Pawlowski et al., 2018). Imaging studies should be performed to investigate olanzapine modulation of related deep brain activity related to appetite.

## Conclusions

In conclusion, our study has shown that appetite is related to olanzapine-induced weight gain and dyslipidemia in drug-naïve first-episode patients with schizophrenia. Assessing appetite changes is an easy and practical way for weight gain prediction, which provides clinicians more time and options for intervention strategies. Early dietary inventions aimed at decreasing appetite and reducing food intake can be helpful for weight control in schizophrenia patients treated with olanzapine.

## Data Availability Statement

The datasets generated for this study are available on request to the corresponding author.

## Ethics Statement

This study was performed in accordance with the Declaration of Helsinki (G.A.o.t.W.M. Association, 2013), and approved by the Ethics Committee of the Second Xiangya Hospital, Central South University. After a complete description of the study to the participants, informed consent was obtained prior to study participation.

## Author Contributions

JH analyzed and interpreted the patient data and was a major contributor in writing the manuscript. G-RH mainly designed and performed the study. YeY, C-CL, J-MX, Y-JL, and X-JP helped in patient recruitment, monitor of the data quality, and document treatment emergent adverse events. YiY helped revised the manuscript. J-PZ guided the study design. RR-W was responsible for the overall content. All authors read and approved the final manuscript.

## Funding

This work was supported by the Key R&D Program Projects, National Science Foundation of China (Grant No.2016YFC1306900) and the National Nature Science Foundation of China (Grant No.81622018 and No. 81901401).

## Conflict of Interest

The authors declare that the research was conducted in the absence of any commercial or financial relationships that could be construed as a potential conflict of interest.

## Supplementary Material

Supplement Figure 1 Velocity of blood glucose and lipid changes in increased appetite group. The mean change values for each patient per month during the study period was analyzed at four time-points: before the month of time to increased appetite (-1M), the month of time to increased appetite (0M), 1 month after the month of time to increased appetite increase (+1M), and 2 months after the month of time to increased appetite (+2M).

Supplement Figure 2 Comparison of insulin and insulin resistance index change velocities of participants with increased appetite in different months. We compared the velocity of participants with an increased appetite within 4 weeks (A) and participants with an increased appetite between 4-8 weeks (B) in the month of time to increased appetite (0M) and 1 month after the month of time to increased appetite (+1M).

Supplement Table 1 Number of patients who had increased initial body weight by more than 7% or 10% at weeks 4, 8, and 12.

Supplement Table 2 Number of patients who had increased initial body weight by more than 7% or 10% at weeks 4, 8, and 12 in patients with increased appetite.

Supplement Table 3 Number of patients who had increased initial body weight by more than 7% or 10% at weeks 4, 8, and 12 in patients with unchanged appetite.

Supplement Table 4 Comparison of numbers of 7% or 10% weight gain between patients with increased appetite and patients with unchanged appetite at weeks 4, 8, and 12.

Supplement Table 5 The analysis of velocity of mean weight gain, glucose, and lipid changes in the appetite increased group at four time-points: before the month of tim to appetite increase (-1M), the month of time to appetite increase (0M), 1 month after the month of time to appetite increase (+1M), and 2 months after the month of time to appetite increase (+2M). ns indicates not significant, * indicates p-value < 0.05, ** indicates p-value < 0.01, *** indicates p-value < 0.001.

Supplement Table 6 Estimates of appetite effects on weight gain and metabolic-related outcome measures from general linear mixed model while controlling for age, duration of illness, and gender.

Supplement Table 7-1 The changes of weight, BMI, Insulin, IRI, and LDL-C per patient during the treatment period in different groups.

Supplement Table 7-2 Estimates of appetite effects on weight gain and metabolic-related outcome measures.

## Abbreviations

SGAs, Second-generation antipsychotics; RCT, Randomized clinical trial; CAFÉ, The Comparison of Atypicals for First Episode; CATIE, The Clinical Antipsychotic Trials in Intervention Effectiveness; DSM-5, The Diagnostic and Statistical Manual of Mental Disorders-Fifth Edition; ECG, Electrocardiogram; PANSS, The Positive and Negative Syndrome Scale; TESS, Treatment Emergent Symptom Scale; ELISA, Enzyme-linked immunosorbent assays; BMI, Body mass index; HDL-C, High-density lipoprotein; LDL-C, Low-density lipoprotein.
