# Corrigendum: Increased Appetite Plays a Key Role in Olanzapine-Induced Weight Gain in First-Episode Schizophrenia Patients

**DOI:** 10.3389/fphar.2020.00878

**Published:** 2020-06-10

**Authors:** Jing Huang, Gang-Rui Hei, Ye Yang, Chen-Chen Liu, Jing-Mei Xiao, Yu-Jun Long, Xing-Jie Peng, Yi Yang, Jing-Ping Zhao, Ren-Rong Wu

**Affiliations:** ^1^Mental Health Institute of the Second Xiangya Hospital, Central South University, Changsha, China; ^2^China National Clinical Research Center on Mental Disorders, Changsha, China; ^3^China National Technology Institute on Mental Disorders, Hunan Key Laboratory of Psychiatry and Mental Health, Changsha, China; ^4^Mental Health Institute, Wenzhou Kangning Hospital, Wenzhou Medical University, Wenzhou, China; ^5^Shanghai Institutes for Biological Sciences, Chinese Academy of Sciences, Shanghai, China

**Keywords:** antipsychotic drugs, appetite, olanzapine, schizophrenia, weight gain

There is an error in the article title. Instead of *Increased Appetite Plays a Key Role in Olanzapine-Induced Weight Gain in*, the title should be *Increased Appetite Plays a Key Role in Olanzapine-Induced Weight Gain in First-Episode Schizophrenia Patients*.

The authors apologize for this error and state that this does not change the scientific conclusions of the article in any way. The original article has been updated.

