# Supplementary figures and images for: Increased Appetite Plays a Key Role in Olanzapine-Induced Weight Gain in First-Episode Schizophrenia Patients
Source: Front Pharmacol. 2020 May 22;11:739. doi: 10.3389/fphar.2020.00739 (PMC7256453; doi:10.3389/fphar.2020.00739)

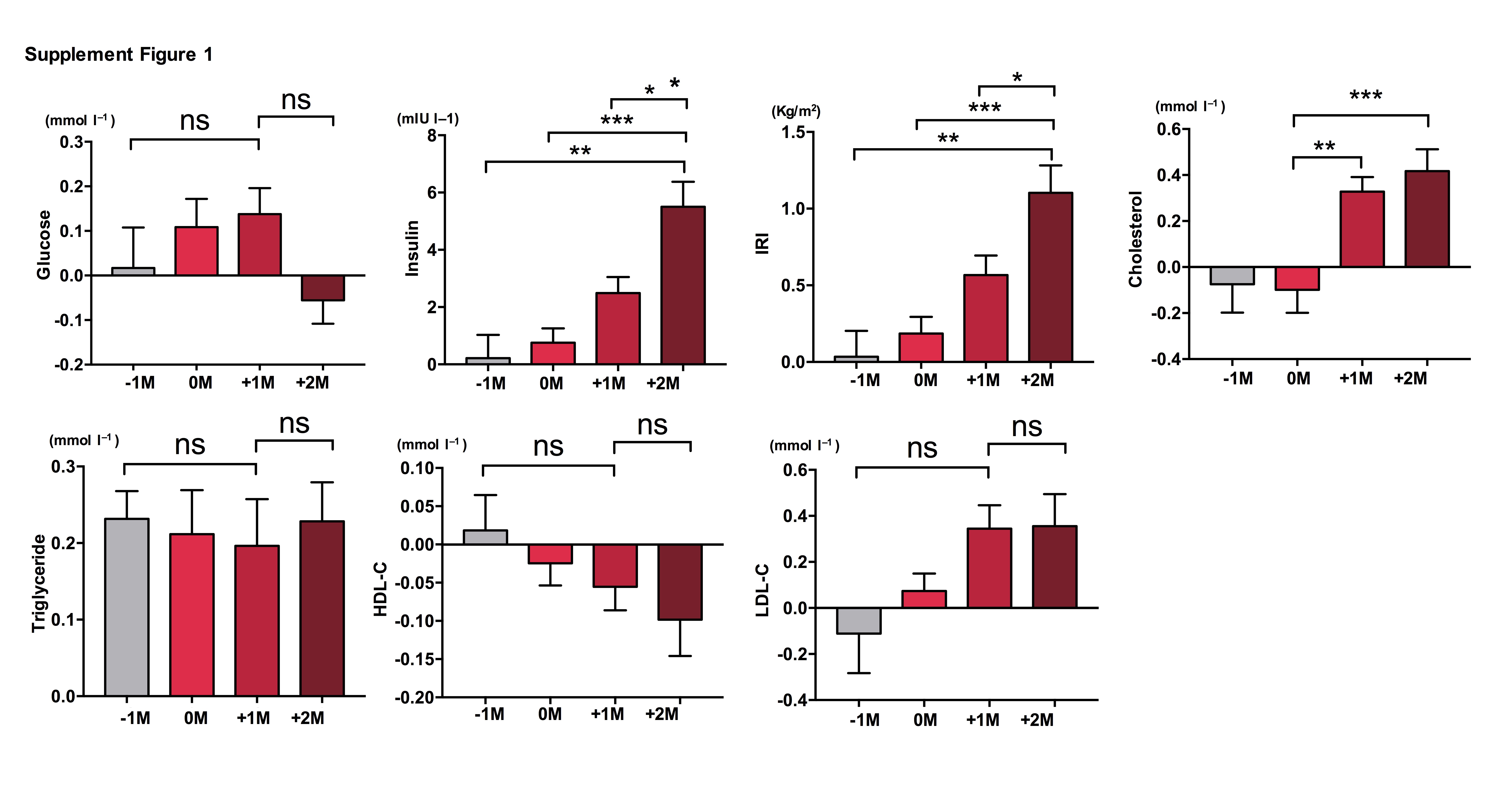

Supplement: Supplement Figure 1 — Velocity of blood glucose and lipid changes in increased appetite group. The mean change values for each patient per month during the study period was analyzed at four time-points: before the month of time to increased appetite (-1M), the month of time to increased appetite (0M), 1 month after the month of time to increased appetite increase (+1M), and 2 months after the month of time to increased appetite (+2M). [file Image_1.jpeg]

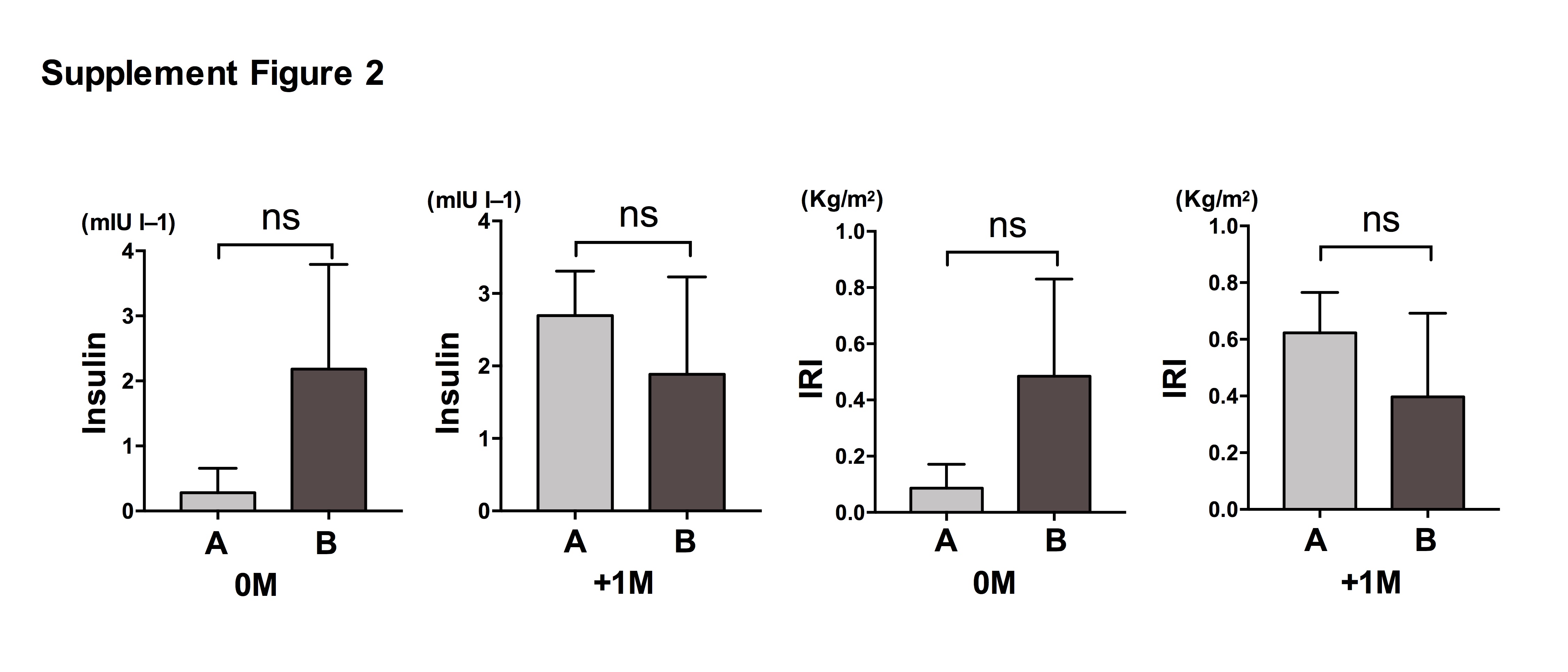

Supplement: Supplement Figure 2 — Comparison of insulin and insulin resistance index change velocities of participants with increased appetite in different months. We compared the velocity of participants with an increased appetite within 4 weeks (A) and participants with an increased appetite between 4-8 weeks (B) in the month of time to increased appetite (0M) and 1 month after the month of time to increased appetite (+1M). [file Image_2.jpeg]
