# Supplementary material for: Increased Appetite Plays a Key Role in Olanzapine-Induced Weight Gain in First-Episode Schizophrenia Patients
Source: Front Pharmacol. 2020 May 22;11:739. doi: 10.3389/fphar.2020.00739 (PMC7256453; doi:10.3389/fphar.2020.00739)
Supplement: Supplement Table 1 — Number of patients who had increased initial body weight by more than 7% or 10% at weeks 4, 8, and 12. [file DataSheet_1.docx]

Supplementary Table 1. Number of patients who had increased initial body weight by more than 7% or 10% at weeks 4, 8, and 12.

| Weeks | n | Number of 7% weight gain | Percentage | p-value | Number of 10% weight gain | Percentage | p-value |
| --- | --- | --- | --- | --- | --- | --- | --- |
| 4 | 33 | 4 | 12.1 | - | 1 | 3.0 | - |
| 8 | 31 | 23 | 74.2 | <0.0001 | 16 | 51.6 | <0.0001 |
| 12 | 31 | 25 | 80.6 | <0.0001 | 19 | 61.3 | <0.0001 |

Supplementary Table 2. Estimates of appetite effect on weight gain and metabolic-related outcome measures from general linear mixed model while controlling for age, duration of illness, and gender.

| Variable and Week | Mean Square | F | P Value |
| --- | --- | --- | --- |
| **Weight** (kg) |  |  |  |
| Week 0 | 0.73 | 0.11 | 0.744 |
| Week 4 | 20.27 | 2.57 | 0.122 |
| Week 8 | 101.85 | 9.54 | 0.005 |
| Week 12 | 183.94 | 17.09 | <0.001 |
| **Body mass index** (kg/m^2^) |  |  |  |
| Week 0 | 0.19 | 0.19 | 0.666 |
| Week 4 | 3.43 | 2.96 | 0.098 |
| Week 8 | 15.89 | 9.78 | 0.004 |
| Week 12 | 27.67 | 16.51 | <0.001 |
| **Fasting glucose** (mmol l^–1^) |  |  |  |
| Week 0 | 0.00 | 0.00 | 0.994 |
| Week 4 | 0.00 | 0.00 | 0.975 |
| Week 8 | 0.19 | 1.55 | 0.225 |
| Week 12 | 0.02 | 0.17 | 0.686 |
| **Fasting insulin** (mIU l^–1^) |  |  |  |
| Week 0 | 0.32 | 0.15 | 0.700 |
| Week 4 | 0.34 | 0.12 | 0.734 |
| Week 8 | 3.54 | 0.35 | 0.559 |
| Week 12 | 167.11 | 6.57 | 0.017 |
| **Insulin resistance index** |  |  |  |
| Week 0 | 0.01 | 0.05 | 0.828 |
| Week 4 | 0.04 | 0.30 | 0.592 |
| Week 8 | 0.36 | 0.78 | 0.385 |
| Week 12 | 7.45 | 6.82 | 0.015 |
| **Triglyceride** (mmol l^–1^) |  |  |  |
| Week 0 | 0.00 | 0.03 | 0.854 |
| Week 4 | 0.09 | 0.83 | 0.371 |
| Week 8 | 0.10 | 0.51 | 0.483 |
| Week 12 | 0.35 | 1.20 | 0.284 |
| **Cholesterol** (mmol l^–1^) |  |  |  |
| Week 0 | 1.28 | 9.08 | 0.006 |
| Week 4 | 1.26 | 5.99 | 0.022 |
| Week 8 | 2.26 | 10.91 | 0.003 |
| Week 12 | 2.62 | 6.25 | 0.019 |
| **HDL-C** (mmol l^–1^) |  |  |  |
| Week 0 | 0.01 | 0.79 | 0.383 |
| Week 4 | 0.02 | 0.78 | 0.384 |
| Week 8 | 0.01 | 0.33 | 0.573 |
| Week 12 | <0.001 | 0.02 | 0.900 |
| **LDL-C** (mmol l^–1^) |  |  |  |
| Week 0 | 0.25 | 2.17 | 0.153 |
| Week 4 | 0.35 | 2.32 | 0.140 |
| Week 8 | 1.72 | 8.17 | 0.008 |
| Week 12 | 3.61 | 13.42 | 0.001 |

Abbreviations: HDL-C, high-density lipoprotein cholesterol; LDL-C, low-density lipoprotein cholesterol.

Supplementary Table 3. Number of patients who had increased initial body weight by more than 7% or 10% at weeks 4, 8, and 12 in patients with increased appetite.

| Weeks | n | Number of 7% weight gain | Percentage | p-value | Number of 10% weight gain | Percentage | p-value |
| --- | --- | --- | --- | --- | --- | --- | --- |
| 4 | 20 | 4 | 20.0 | - | 1 | 0.5 | - |
| 8 | 24 | 21 | 87.5 | <0.0001 | 16 | 66.7 | <0.0001 |
| 12 | 24 | 22 | 91.7 | <0.0001 | 19 | 79.2 | <0.0001 |

Supplementary Table 4. Number of patients who had increased initial body weight by more than 7% or 10% at weeks 4, 8, and 12 in patients with unchanged appetite.

| Weeks | n | Number of 7% weight gain | Percentage | p-value | Number of 10% weight gain | Percentage | p-value |
| --- | --- | --- | --- | --- | --- | --- | --- |
| 4 | 13 | 0 | 0 | - | 0 | 0 | - |
| 8 | 7 | 2 | 28.6 | 0.0422 | 0 | 0 | - |
| 12 | 7 | 3 | 42.9 | 0.0105 | 0 | 0 | - |

Supplementary Table 5. Comparison of numbers of 7% or 10% weight gain between patients with increased appetite and patients with unchanged appetite at weeks 4, 8, and 12.

| Number of 7% weight gain | | | |  | Number of 10% weight gain | | |
| --- | --- | --- | --- | --- | --- | --- | --- |
| Weeks | $\chi2$ | df | p-value |  | $\chi2$ | df | p-value |
| 4 | 1.225464 | 1 | 0.268291 |  | 0.277644 | 1 | 0.598249 |
| 8 | 9.828901 | 1 | 0.001718 |  | 9.644444 | 1 | 0.001899 |
| 12 | 8.271587 | 1 | 0.004027 |  | 14.315972 | 1 | 0.000155 |

Supplementary Table 6. The analysis of velocity of mean weight gain, glucose, and lipid changes in the appetite increased group at four time-points: before the month of time to appetite increase (-1M), the month of time to appetite increase (0M), 1 month after the month of time to appetite increase (+1M), and 2 months after the month of time to appetite increase (+2M).

Weight gain

| Comparison | Mean difference | | 95% CI of difference | Significance | Adjusted *P* value |
| --- | --- | --- | --- | --- | --- |
| -1M vs. 0M | -1.77 | -3.91 to 0.38 | | ns | 0.1417 |
| -1M vs. +1M | -2.94 | -5.10 to -0.78 | | ** | 0.0035 |
| -1M vs. +2M | -0.27 | -2.50 to 1.97 | | ns | 0.9891 |
| 0M vs. +1M | -1.17 | -2.51 to 0.17 | | ns | 0.1085 |
| 0M vs. +2M | 1.50 | 0.05 to 2.95 | | * | 0.0399 |
| +1M vs. +2M | 2.67 | 1.20 to 4.15 | | **** | <0.0001 |

Mean weight gain per month at different time-points

-1M (mean 1.37 ± 1.23 (range -0.5–2.4)

0M (mean 3.13 ± 1.36 (range 0–6.8)

1M (mean 4.30± 2.43 (range -0.2–10.2)

2M (mean 1.63 ± 1.47 (range -0.8–5.3)

BMI

| Comparison | Mean difference | 95% CI of difference | Significance | Adjusted *P* value |
| --- | --- | --- | --- | --- |
| -1M vs. 0M | -0.69 | -1.55 to 0.17 | ns | 0.1587 |
| -1M vs. +1M | -1.15 | -2.02 to -0.28 | ** | 0.0047 |
| -1M vs. +2M | -0.11 | -1.00 to 0.79 | ns | 0.9896 |
| 0M vs. +1M | -0.46 | -0.99 to 0.08 | ns | 0.1253 |
| 0M vs. +2M | 0.59 | 0.00 to 1.17 | * | 0.0481 |
| +1M vs. +2M | 1.04 | 0.45 to 1.64 | **** | <0.0001 |

Insulin

| Comparison | Mean difference | 95% CI of difference | Significance | Adjusted *P* value |
| --- | --- | --- | --- | --- |
| -1M vs. 0M | -0.54 | -4.00 to 2.92 | ns | 0.9762 |
| -1M vs. +1M | -2.28 | -5.74 to 1.19 | ns | 0.3157 |
| -1M vs. +2M | -5.28 | -8.86 to -1.71 | ** | 0.0013 |
| 0M vs. +1M | -1.73 | -3.92 to 0.46 | ns | 0.1681 |
| 0M vs. +2M | -4.74 | -7.11 to -2.38 | **** | <0.0001 |
| +1M vs. +2M | -3.01 | -5.37 to -0.64 | ** | 0.0070 |

IRI

| Comparison | Mean difference | 95% CI of difference | Significance | Adjusted *P* value |
| --- | --- | --- | --- | --- |
| -1M vs. 0M | -0.15 | -0.90 to 0.60 | ns | 0.9497 |
| -1M vs. +1M | -0.53 | -1.28 to 0.21 | ns | 0.2452 |
| -1M vs. +2M | -1.07 | -1.84 to -0.30 | ** | 0.0027 |
| 0M vs. +1M | -0.38 | -0.85 to 0.09 | ns | 0.1548 |
| 0M vs. +2M | -0.92 | -1.43 to -0.41 | **** | <0.0001 |
| +1M vs. +2M | -0.54 | -1.05 to -0.03 | * | 0.0349 |

Glucose

| Comparison | Mean difference | 95% CI of difference | Significance | Adjusted *P* value |
| --- | --- | --- | --- | --- |
| -1M vs. 0M | -0.10 | -0.43 to 0.24 | ns | 0.8870 |
| -1M vs. +1M | -0.12 | -0.45 to 0.21 | ns | 0.7753 |
| -1M vs. +2M | 0.07 | -0.27 to 0.42 | ns | 0.9455 |
| 0M vs. +1M | -0.03 | -0.24 to 0.18 | ns | 0.9833 |
| 0M vs. +2M | 0.16 | -0.06 to 0.39 | ns | 0.2395 |
| +1M vs. +2M | 0.19 | -0.04 to 0.42 | ns | 0.1247 |

Triglyceride

| Comparison | Mean difference | | 95% CI of difference | Significance | Adjusted *P* value |
| --- | --- | --- | --- | --- | --- |
| -1M vs. 0M | | 0.02 | -0.30 to 0.34 | ns | 0.9983 |
| -1M vs. +1M | | 0.04 | -0.28 to 0.35 | ns | 0.9910 |
| -1M vs. +2M | | 0.00 | -0.32 to 0.33 | ns | >0.9999 |
| 0M vs. +1M | | 0.02 | -0.18 to 0.22 | ns | 0.9970 |
| 0M vs. +2M | | -0.02 | -0.23 to 0.20 | ns | 0.9970 |
| +1M vs. +2M | | -0.03 | -0.25 to 0.18 | ns | 0.9796 |

Cholesterol

| Comparison | Mean difference | 95% CI of difference | Significance | Adjusted *P* value |
| --- | --- | --- | --- | --- |
| -1M vs. 0M | 0.02 | -0.46 to 0.51 | ns | 0.9992 |
| -1M vs. +2M | -0.49 | -0.99 to 0.00 | ns | 0.0532 |
| 0M vs. +1M | -0.43 | -0.73 to -0.12 | ** | 0.0025 |
| 0M vs. +2M | -0.52 | -0.85 to -0.19 | *** | 0.0006 |
| +1M vs. +2M | -0.09 | -0.42 to 0.24 | ns | 0.8893 |

HDL-C

| Comparison | Mean difference | 95% CI of difference | Significance | Adjusted *P* value |
| --- | --- | --- | --- | --- |
| -1M vs. 0M | 0.04 | -0.15 to 0.24 | ns | 0.9356 |
| -1M vs. +1M | 0.07 | -0.12 to 0.27 | ns | 0.7447 |
| -1M vs. +2M | 0.12 | -0.08 to 0.32 | ns | 0.4165 |
| 0M vs. +1M | 0.03 | -0.09 to 0.15 | ns | 0.9091 |
| 0M vs. +2M | 0.07 | -0.06 to 0.21 | ns | 0.4568 |
| +1M vs. +2M | 0.04 | -0.09 to 0.17 | ns | 0.8256 |

LDL-C

| Comparison | Mean difference | 95% CI of difference | | Significance | Adjusted *P* value |
| --- | --- | --- | --- | --- | --- |
| -1M vs. 0M | -0.19 | -0.74 to 0.37 | | ns | 0.8169 |
| -1M vs. +1M | -0.46 | -1.01 to 0.10 | | ns | 0.1439 |
| -1M vs. +2M | -0.47 | -1.04 to 0.11 | | ns | 0.1501 |
| 0M vs. +1M | -0.27 | -0.64 to 0.10 | | ns | 0.2197 |
| 0M vs. +2M | -0.28 | -0.68 to 0.11 | | ns | 0.2464 |
| +1M vs.+2M | -0.01 | | -0.41 to 0.38 | ns | 0.9999 |

Supplementary Table 7-1. The changes of weight, BMI, Insulin, IRI, and LDL-C per patient during the treatment period in different groups.

| Indicators | Total sample | patients with increased appetite | patients with unchanged appetite | Mediating effect |
| --- | --- | --- | --- | --- |
| Weight (kg) | 7.92 | 9.10 | 3.86 | 66.2% |
| BMI (kg/m2) | 3.05 | 3.55 | 1.35 | 72.1% |
| Insulin (mIU l–1) | 6.03 | 7.43 | 1.21 | 103.1% |
| IRI | 1.30 | 1.59 | 0.30 | 99.3% |
| LDL-C (mmol l–1) | 0.48 | 0.08 | 0.60 | 106.7% |

Supplement Table 7-2. Estimates of appetite effects on weight gain and metabolic-related outcome measures.

|  | Weight | BMI | Glucose | Insulin | *IRI* | Triglyceride | Cholesterol | HDL-C | LDL-C |
| --- | --- | --- | --- | --- | --- | --- | --- | --- | --- |
| β | 0.67 | 0.63 | 0.10 | 0.49 | 0.51 | 0.30 | 0.39 | -0.01 | 0.61 |
| P | 0.0003 | 0.0004 | 0.6170 | 0.0189 | 0.0149 | 0.1478 | 0.1357 | 0.9665 | 0.0035 |
